# Supplementary material for: Estimating the case fatality ratio for COVID-19 using a time-shifted distribution analysis
Source: Epidemiol Infect. 2021 Jul 19;149:e197. doi: 10.1017/S0950268821001436 (PMC8438516; doi:10.1017/S0950268821001436)
Supplement: Supplementary file 1 [file S0950268821001436sup001.docx]

**Supplementary Material**

**Appendix 1: Overlay of daily COVID-19 cases (time-shifted and scaled) and deaths for many countries**

Time-shifted distribution analysis gives two parameters: the time delay between case and death distributions and the case fatality risk, which acts as a linear scaling factor between time-shifted cases and deaths. The following plots overlay the daily time-shifted and scaled case distribution with the daily death distribution for each of the countries listed in Table 1, demonstrating excellent correlations over diverse outbreak characteristics, even where data are noisy.

**
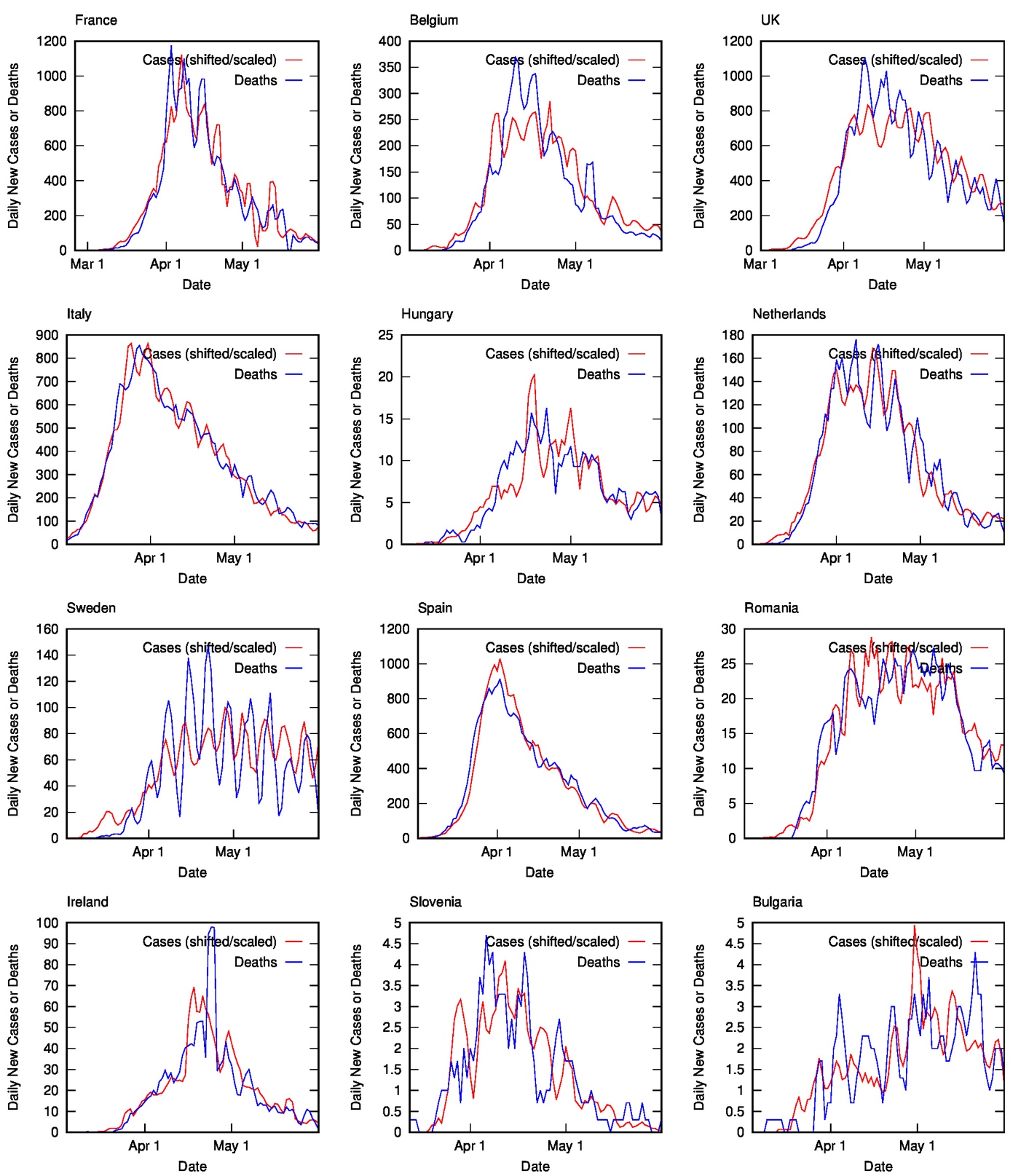
**

**
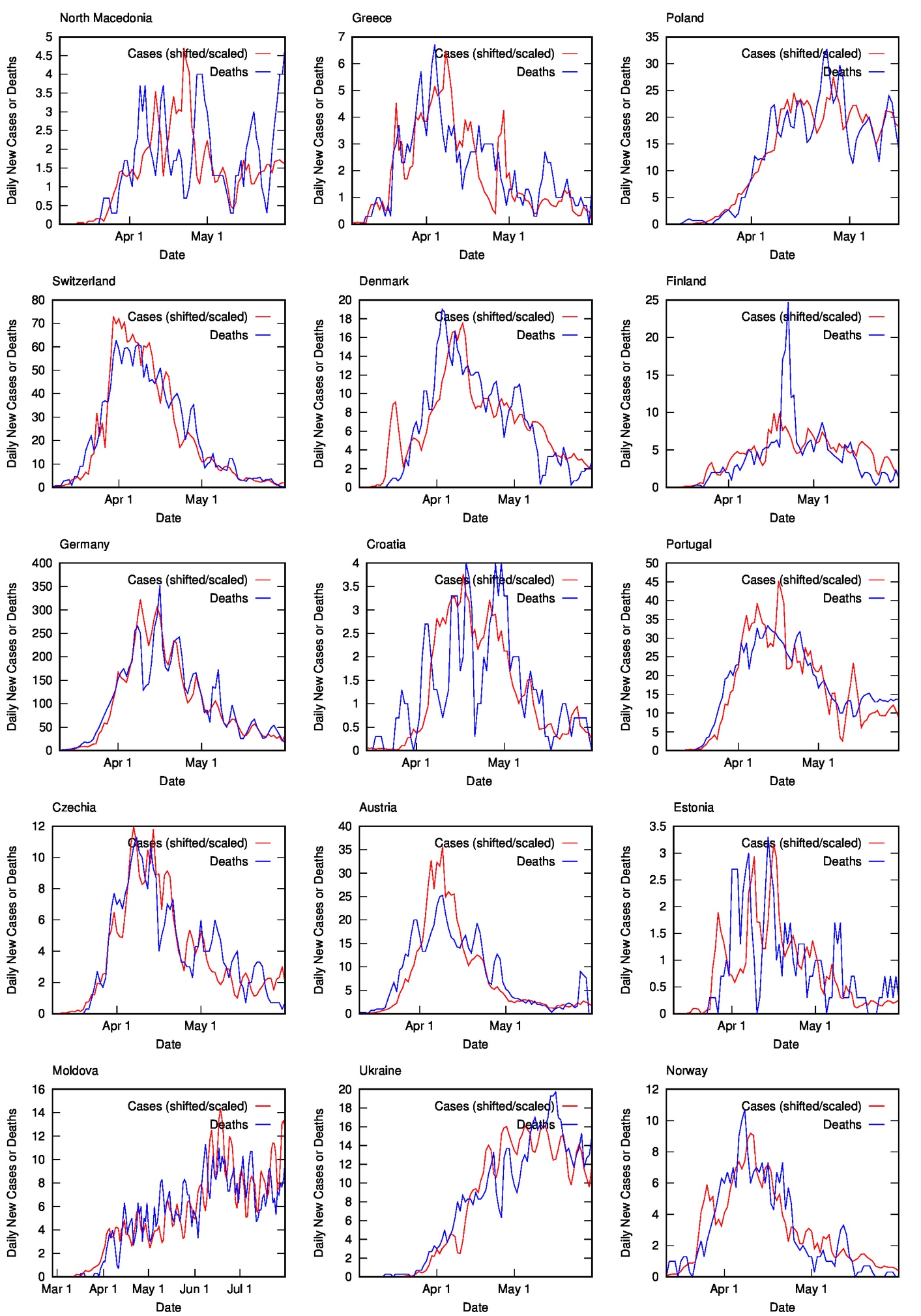

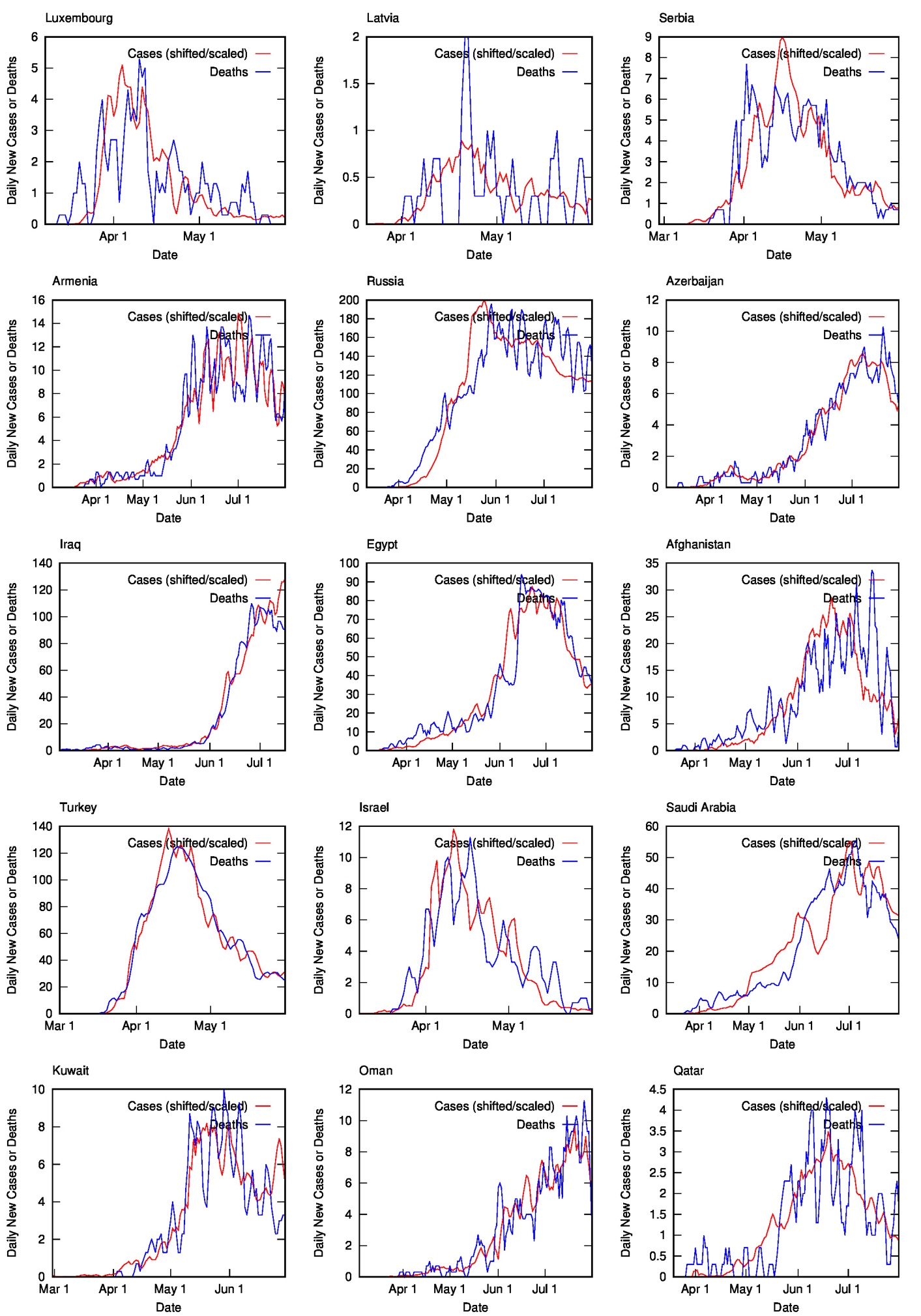

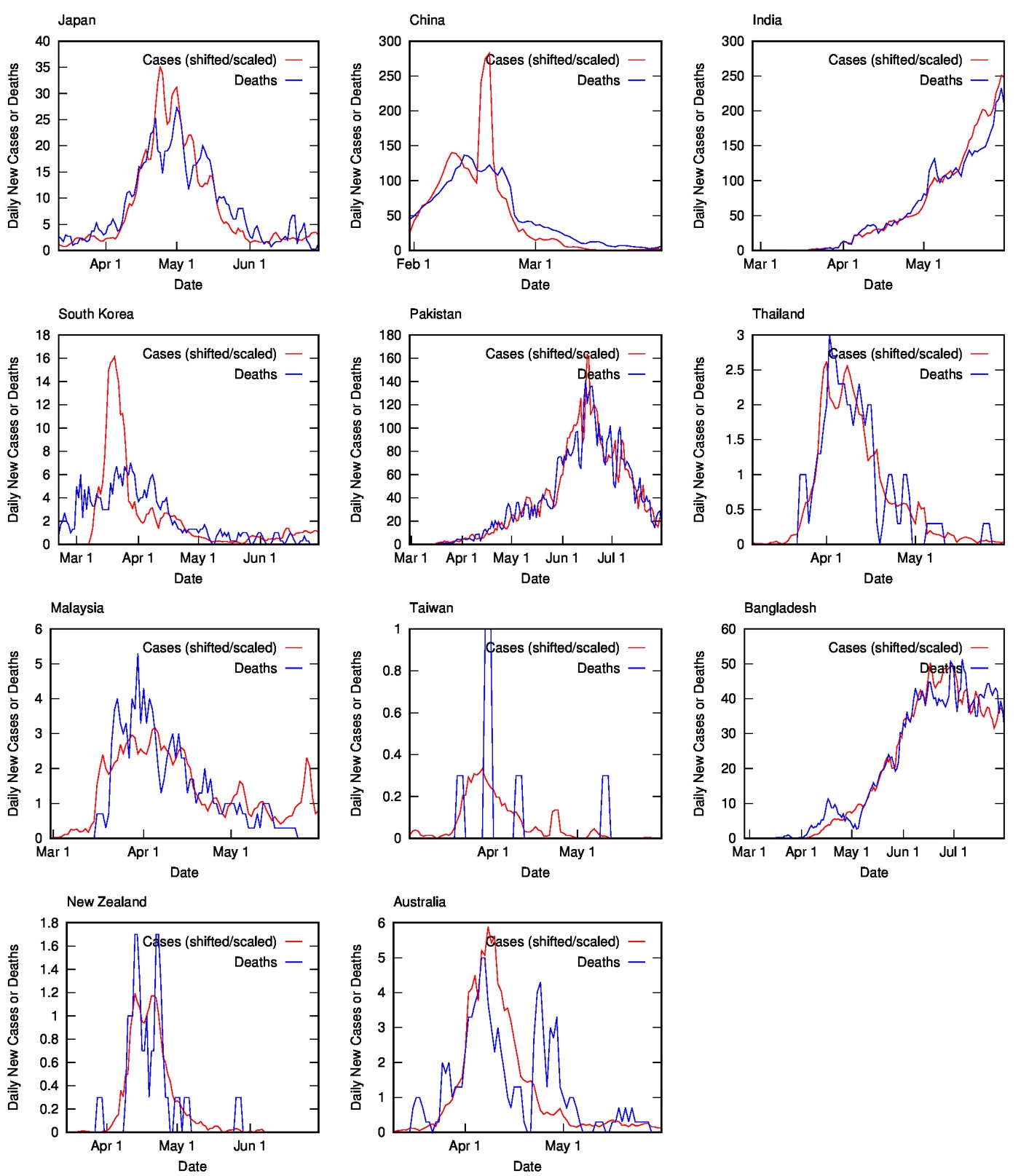

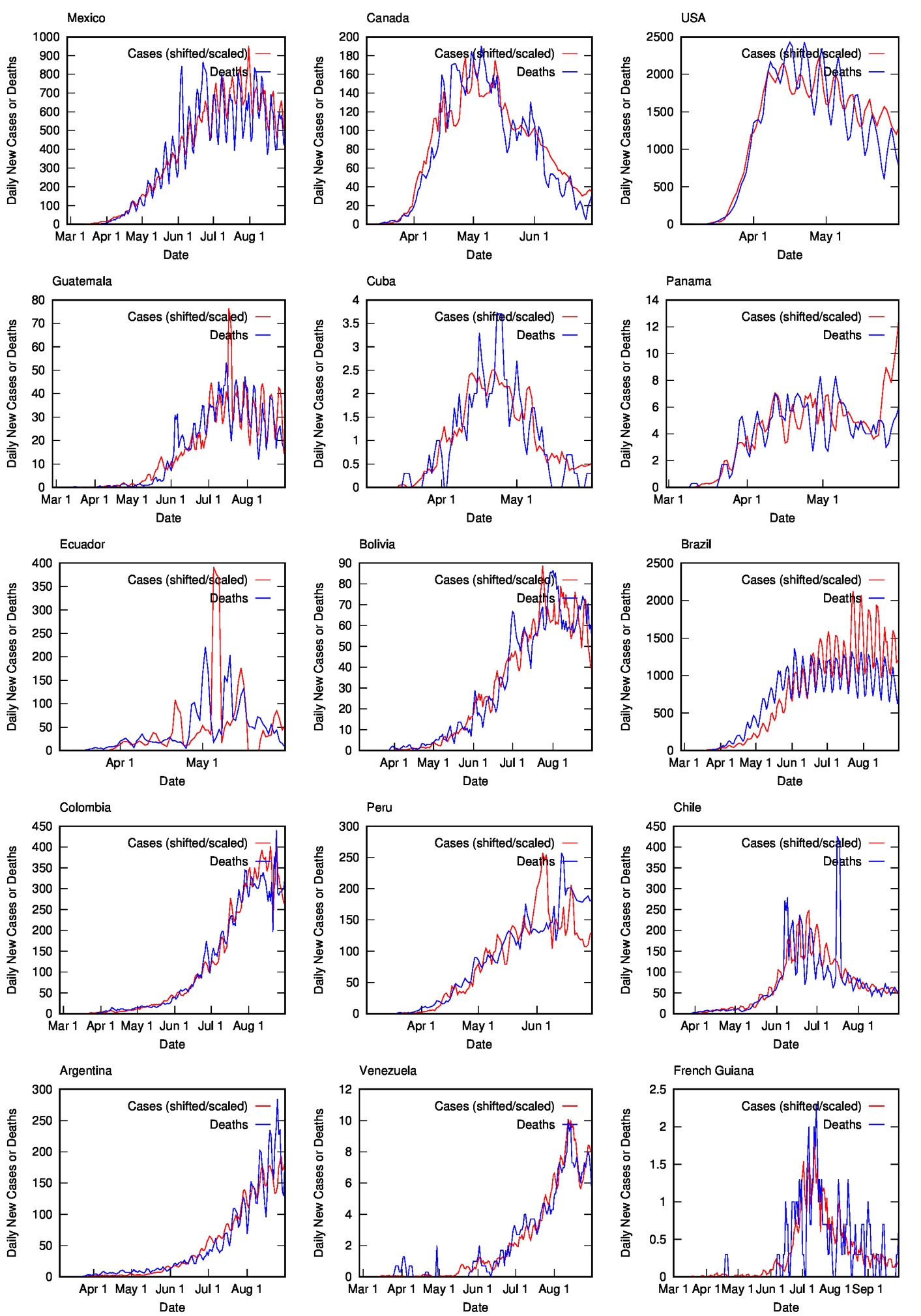
**

**
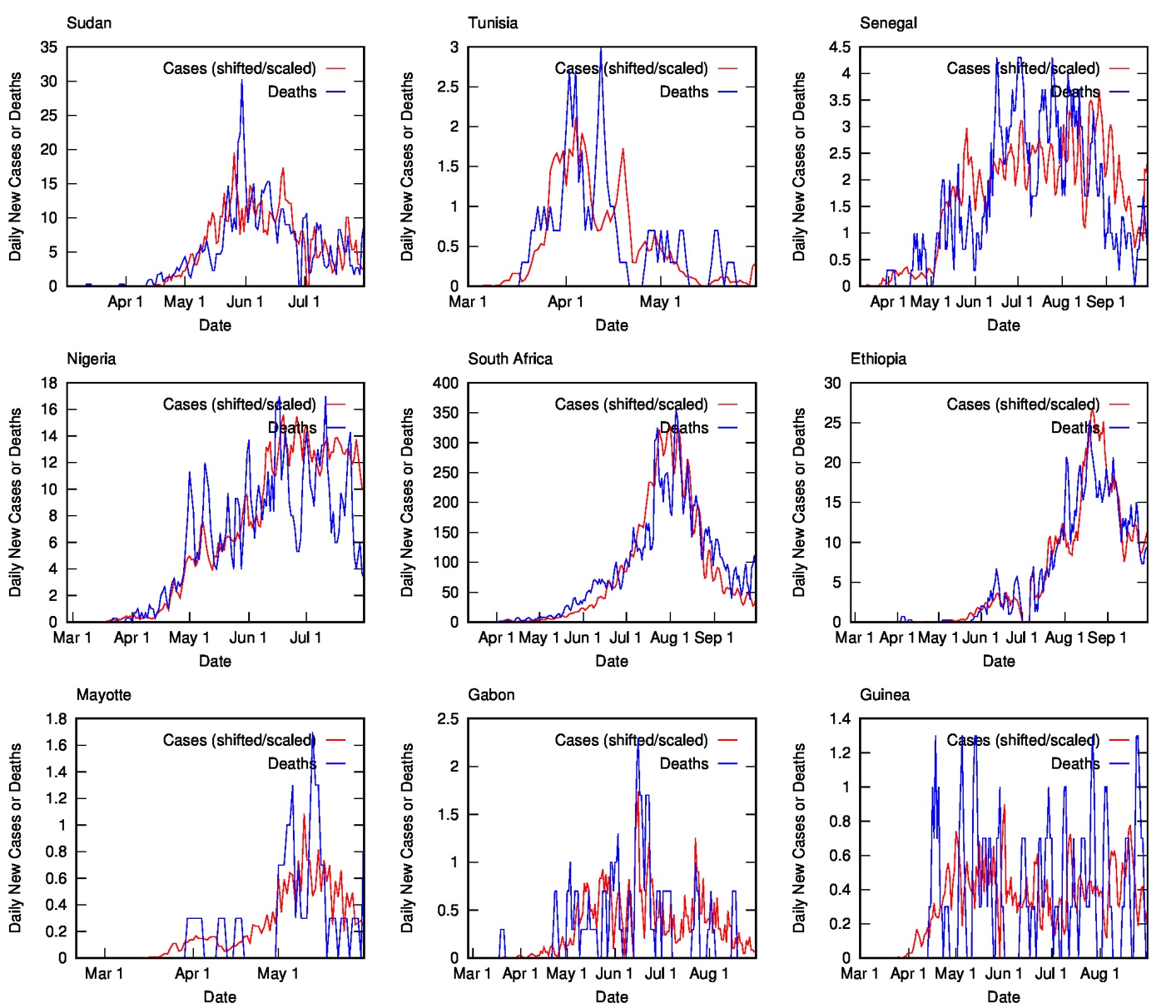
**
